# COVID-19: Molecular and Cellular Response

**DOI:** 10.3389/fcimb.2021.563085

**Published:** 2021-02-11

**Authors:** Shamila D. Alipoor, Esmaeil Mortaz, Hamidreza Jamaati, Payam Tabarsi, Hasan Bayram, Mohammad Varahram, Ian M. Adcock

**Affiliations:** ^1^ Molecular Medicine Department, Institute of Medical Biotechnology, National Institute of Genetic Engineering and Biotechnology (NIGEB), Tehran, Iran; ^2^ Clinical Tuberculosis and Epidemiology Research Center, National Research Institute of Tuberculosis and Lung Diseases, Shahid Beheshti University of Medical Sciences, Tehran, Iran; ^3^ Department of Immunology, School of Medicine, Shahid Beheshti University of Medical Sciences, Tehran, Iran; ^4^ Chronic Respiratory Diseases Research Center, National Research Institute of Tuberculosis and Lung Diseases (NRITLD), Shahid Beheshti University of Medical Sciences, Tehran, Iran; ^5^ Department of Pulmonary Medicine, Koc University School of Medicine, Koc University Research Center for Translational Medicine (KUTTAM), Istanbul, Turkey; ^6^ Mycobacteriology Research Center, National Research Institute of Tuberculosis and Lung Diseases (NRITLD), Shahid Beheshti University of Medical Sciences, Tehran, Iran; ^7^ National Heart and Lung Institute, Imperial College London and the NIHR Imperial Biomedical Research Centre, London, United Kingdom; ^8^ Priority Research Centre for Asthma and Respiratory Disease, Hunter Medical Research Institute, University of Newcastle, Newcastle, NSW, Australia

**Keywords:** COVID-19, cytokines, IL-6, IL-8, ACE2

## Abstract

In late December 2019, a vtiral pneumonia with an unknown agent was reported in Wuhan, China. A novel coronavirus was identified as the causative agent. Because of the human-to-human transmission and rapid spread; coronavirus disease 2019 (COVID-19) has rapidly increased to an epidemic scale and poses a severe threat to human health; it has been declared a public health emergency of international concern (PHEIC) by the World Health Organization (WHO). This review aims to summarize the recent research progress of COVID-19 molecular features and immunopathogenesis to provide a reference for further research in prevention and treatment of SARS coronavirus2 (SARS-CoV-2) infection based on the knowledge from researches on SARS-CoV and Middle East respiratory syndrome-related coronavirus (MERS-CoV).

## Introduction

Coronavirus disease 2019 (COVID-19) is caused by a member of the Coronaviridae family. It was initially reported in Wuhan, China and has expanded rapidly in the world and is now at pandemic level (Lippi and Plebani, 2020). The novel β-CoV was named as “SARS-CoV-2” by the International Virus Classification Commission (Wu F. et al., 2020).

Coronaviruses are enveloped positive-stranded RNA viruses, which replicate in the host cell cytoplasm. They possess a 5’ capped RNA and also contain the longest RNA among all RNA viruses with a length of ~30kbp containing 14 open reading frames (ORFs) (Marra et al., 2003; Perlman and Netland, 2009). Coronaviruses are classified into four genera (α, β, γ, and δ) based on phylogeny (Su et al., 2016).

The first human coronavirus was described in 1965 from patients with the common cold for which there is still no vaccine. Counting SARS coronavirus2 (SARS-Cov2), there are currently seven human coronaviruses strains known to infect humans which belong to the α and β coronavirus genera (**Table 1**) (Su et al., 2016; Rithanya and Brundha, 2020).

**Table 1:** Human Corona virus overview.

Name	Discovery/location	Group	Receptor	Symptom
HCOV-229E	1966	α	Aminopeptidase N (hAPN, CD13)	Common cold
HCoV-OC43	1967	ß	9-*O*-acetylated sialic acid	Common cold
SARS-CoV	2003/china	ß	ACE2/CD209L	Severe respiratory illness
HCoV-NL63	2004/Netherlands	α	ACE2	Lower respiratory tract infection, croup and bronchiolitis in children and immunocompromised patients
HCoV-HKU1	2005/HongKong	ß	9-*O*-acetylated sialic acid	Chronic pulmonary disease in Hong Kong
MERS-CoV	2012/Middle East	ß	DPP4	Severe respiratory illness
Sars-Cov2	2019/China	ß	ACE2	Severe respiratory illness

Coronaviruses gained notoriety with the outbreak of Severe Acute Respiratory Syndrome (SARS) in 2002-2003. This led to the isolation and identification of HCoV-NL63 and HCoV-HKU1. Further, the emergence of the middle east respiratory syndrome coronavirus (MERS-CoV) in 2012 revealed that these pathogens frequently cross the species and can pose a serious risk to human health.

Bats are the main reservoirs of a large variety of viruses especially human α- and β-coronaviruses (Hu et al., 2015). Some SARS-like coronavirus (SL-CoVs) isolated from the bats have high genomic sequence similarity and receptor usage compared to SARS-CoV. This suggests that the spread of a bat coronavirus to man presents a major global health risk (Ge et al., 2013; Menachery et al., 2015; Yang X. L. et al., 2015). In the case of the novel β-CoV strain, genome sequencing revealed (Lu et al., 2020) the new virus has 81% identity with the sequence of the bat-derived SARS.

The majority of COVID 19 cases (about 80%) are asymptomatic or show mild symptoms but a low percentage experience severe respiratory failure (Surveillances, 2020). Interestingly, the viral load in asymptomatic patients was similar to that in symptomatic patients. In a study in china it was reported that in people with normal CT scans and no clinical symptoms, who were in close contact with confirmed virus-positive patients; nasal and throat swab tests were positive on days 7, 10, and 11 after contact (Zou et al., 2020). Furthermore, SARS-CoV-2 RNA was detectable in stool, saliva and urine samples as well as in gastrointestinal tissue in patients with COVID-19 in China. Thus, the digestive tract should also be considered as a route of infection (Xiao et al., 2020). These findings emphasize the potential of viral transmission of asymptomatic patients and indicate the urgent need for strategies revolving around case detection and isolation (Surveillances, 2020).

Since Dec 2019, when the first case of disease was reported, there have been over 80 M confirmed cases and 1.7 M deaths have been reported across 235 countries (https://www.who.int/emergencies/diseases/novel-coronavirus-2019). Given its properties and rapid spread, there is an emergent need to expand our knowledge of the molecular features and immune pathogenesis of COVID-19. This review summarizes recent findings on the potential mechanisms and clinical features of COVID-19 and relies on our knowledge of SARS-CoV and MERS-CoV. This work aims to provide a reference for further research in the prevention and the treatment of SARS-CoV-2 infection

## Virology and Genome

The SARS-CoV-2 genome consist of 29,903nt (nucleotides) and has been assigned GenBank accession number MN908947. RNA from the virus is closely related to two bat derived SARS-like coronaviruses SL-CoVZC45 and SL-CoVZXC21, with a nucleotide identity of 88.1%, but is more distant from SARS-CoV (about 79%) and MERS-CoV (about 50%) (Lu et al., 2020; Wu F. et al., 2020).

The genome of SARS-Cov-2, similar to other CoVs, contains ten open reading frames (ORFs). The first ORF covers two-thirds of the viral RNA, which encodes polypeptides of the viral replicase-transcriptase complex (Fehr and Perlman, 2015). The remaining ORFs translate four main structural proteins: spike (S), envelope (E), nucleocapsid (N), and membrane (M) proteins. The genome is packaged into a helical nucleocapsid surrounded by a host-derived lipid bilayer (Fehr and Perlman, 2015).

## Structural Determinants in the Pathogenicity of SARS-Cov2

A key to tackling the new pandemic is a clear understanding of the factors that determine the virus pathogenicity including the mechanism underlying receptor recognition, cell entry, and the processing of viral proteins inside cells.

### Virus Receptor

Receptor recognition is crucial in viral infectivity, pathogenesis and cell tropism and can be considered as the primary determinant of virus pathogenicity.

Corona viruses use a variety of receptors to enter the cells. Dipeptidyl protease 4 (DPP4) or CD26 is used by MERS-CoV (Wang et al., 2013) and angiotensin converting enzyme 2 (ACE2) by SARS-CoV (Jeffers et al., 2004) and NL63-Cov (Hofmann et al., 2005). CD209L is also used by SARS-CoV as an alternative receptor (Jeffers et al., 2004) (**Table 1**). Based on viral genome analysis of the new virus and high similarity to SARS-CoV, it is likely that SARS-CoV-2 uses ACE2 as its receptor for cellular invasion (Xu H. et al., 2020; Wan Y. et al., 2020). Further evidence for ACE2 being the SARS-CoV-2 receptor is provided by the fact that Baby Hamster Kidney fibroblasts (BHk) cells transfected with human ACE2 become susceptible to SARS-CoV-2 infection (Walls et al., 2020).

The ACE2 enzyme is involved in the renin–angiotensin–aldosterone system (RAAS) activation. The RAAS includes a cascade of vasoactive peptides that regulate the blood volume and systemic vascular resistance in a prolonged manner (Dronavalli et al., 2008). ACE2 is an exo-peptidase that catalyzes the conversion of angiotensin (Ang I) I to the nonapeptide Ang-(1-9) or the conversion of angiotensin II to the heptapeptide Ang-(1-7) (Dronavalli et al., 2008).

Previous studies demonstrated a positive correlation between the expression pattern of ACE2 and the infectivity of SARS-CoV. In addition, some ACE2 variants interact with the SARS-CoV-2 or NL635 S-proteins with a lower-affinity (Mathewson et al., 2008). Therefore, the pattern of ACE2 expression in different tissues can determine tropism, susceptibility, symptoms, and outcome of SARS-CoV-2 infection (Chen J. et al., 2020). ACE2 is expressed on the mucosa of oral cavity and is highly enriched in epithelial cells of the tongue which highlights the role of the oral cavity for infection with SARS-CoV-2 (Xu H. et al., 2020). ACE2 is also highly expressed in vascular endothelial cells of the heart and the kidney, and it affects cardiac function (Luft, 2014; Badae et al., 2019).

COVID-19 has a higher mortality rate in males compared to females (Xie et al., 2020) despite both genders having a similar infection rate (The Sex, Gender, and COVID-19 Project, 2020). There are differences in the reported male-biased mortality between countries ranging from 59%–75% of total mortality (Pradhan and Olsson, 2020; Jin et al., 2020). Biological (immune responses) and behavioral factors (e.g., smoking, mask wearing and other lifestyle habits) may be responsible for placing men at a greater risk of infection by SARS-CoV-2 or the consequences of COVID-19 infection (Galasso et al., 2020). However, in a few countries such as India, Nepal, Vietnam, and Slovenia the COVID-19 case fatality rate is higher in women than men (The Sex, Gender, and COVID-19 Project, 2020). These differential findings may be due to the incomplete data in the case identification by sex or demographic factors such as co-morbidities that increase the health risk for women in these regions (Dehingia and Raj, 2020).

A recent study reported that Asian males have higher expression of ACE2 (Zhao et al., 2020). It was also previously reported that German cases showed mild clinical symptoms for SARS without severe illness (Cao et al., 2020). However, the pattern of ACE2 expression and function in different populations remains to be investigated (Cao et al., 2020). For example, the rate of COVID 19 related death is significantly higher among the Afro-Caribbean and South-East Asian people. Data to date indicates that the confirmed cases of COVID-19 in black counties is more than 3-fold-, and the rate of death is 6-fold-, higher than that in white counties in the USA (Yancy, 2020; Thebault R and Williams, 2020). This stark difference in the rate of disease and outcome in the black population may be explained in part, but not fully, by concomitant comorbidities (Giudicessi et al., 2020). Comorbidities such as hypertension, metabolic syndrome, diabetes, and cardiovascular disease (CVD) are the main risk factors for worse outcome and mortality in patients with COVID-19 (Bansal, 2020) and these are prevalent in Afro-Caribbean and south-East Asian populations (Owczarek et al., 2018).

### Spike Glycoprotein (S Protein)

The viral spike glycoprotein (S protein) binds to the host cellular receptor and is therefore, considered as the main determinant of cell tropism and pathogenesis (Kuba et al., 2010). In corona viruses; the spike protein is a large transmembrane protein and is highly glycosylated. Spike proteins assemble as homo-trimers on the virion surface to form a crown-like appearance or “corona” (Belouzard et al., 2012).

**Figure 1:**
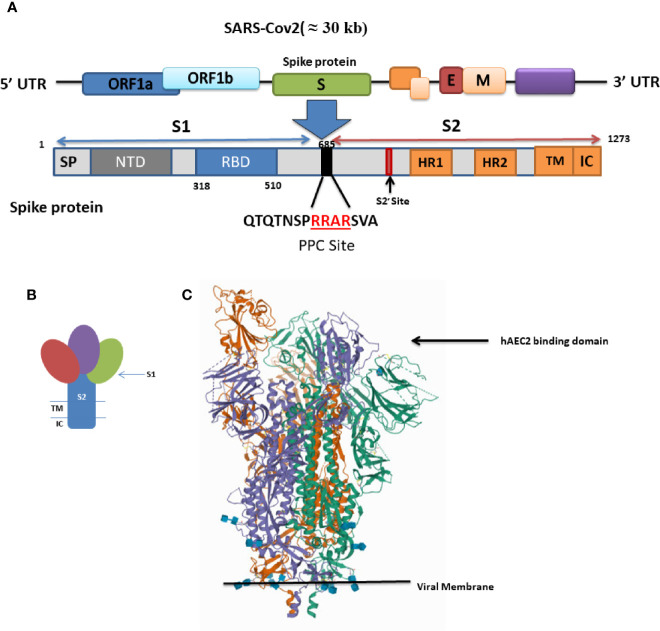
**(A)** Schematic of genome encoding spike protein in SARS-CoV2. SP, signal peptide; NTD, N-terminal domain; RBD, receptor-binding domain; RBM, receptor-binding motif; FP, fusion peptide; HR1 and HR2, heptad repeat regions 1 and 2; TM, transmembrane; CP, cytoplasmic domain. **(B)** Schematic drawing of the structure of coronavirus spike. S1, receptor-binding subunit (homotrimers); S2, membrane fusion subunit; TM, transmembrane anchor; IC, intracellular tail. **(C)** SARS-CoV-2 spike ectodomain structure (open state) (details provided at https://www.rcsb.org/structure/6VYB).

The S protein is composed of an extracellular and a transmembrane domain (TM), as well as a short cytoplasmic tail region (CP) (**Figure 1A**). The extracellular domain of the S protein is composed of two subunits (S1 and S2) which are responsible for host-cell receptor recognition and membrane fusion, respectively. S1 binds to ACE2 *via* the receptor-binding domain (RBD) (Bosch et al., 2003).

A three-dimensional (3-D) atomic-scale map of the RBD of SARS-CoV-2 in complex with human ACE2 has been obtained (https://www.rcsb.org/structure/6VYB) (**Figure 1B**) (Shang et al., 2020a). This analysis shows that the RBD of the SARS CoV-2 S protein is more compact and binds human ACE-2 four times more strongly than the SARS-CoV S protein (Shang et al., 2020a). The RBD is the most variable part of the coronavirus genome. Six RBD amino acids are critical for ACE2 binding (Tai et al., 2020). Five of these six residues differ between SARS-CoV-2 and SARS-CoV, which accounts for the higher binding affinity of the SARS-CoV-2 S protein for human ACE2 (Andersen et al., 2020).

Even though the SARS-CoV-2 RBD has a higher binding affinity than SARS-CoV RBD for human ACE2, the binding affinity of the entire SARS-CoV-2 S protein for human ACE2 is lower than, or comparable to, that of the SARS-CoV S protein (Shang et al., 2020b). This paradox can be explain based on the dynamic state of the RBD. Cryo-electron microscopy studies captured two states of the RBD: either buried (lying state) or exposed (standing state), illustrating an inherently flexible RBD readily recognized by its receptor (Yuan et al., 2017) (**Figure 1C**).

The RBD in the SARS-CoV S protein is mostly in the standing-up state, however, it is mostly in the lying-down state in SARS-CoV-2 and is not exposed and unable to bind ACE2. Thus, although the SARS-CoV-2 RBD is more potent, it is less exposed than the SARS-CoV RBD and the overall entry efficiencies of SARS-CoV-2 and SARS-CoV are comparable (Shang et al., 2020b). The hidden RBD may also be responsible for inefficient immune response and prolonged recovery time as well as for the long incubation period. Many SARS-CoV-2 patients develop low levels of neutralizing antibodies and suffer prolonged illness.

### Proteolytic Processing

Host protease activation is the other significant determinant of coronavirus infection and pathogenesis. Coronavirus entry is tightly regulated by the expression and activation of host proteases.

The presence of a proteolytic cleavage site within the S protein mediates membrane fusion and viral infectivity. Sequence variation around this cleavage site impacts severely on cellular tropism and pathogenesis of CoVs (Kido et al., 2012; Coutard et al., 2020). In the case of H5N1 hemagglutinin HA, insertion of a multi-basic motif created a typical furin-like cleavage site in the S protein which was responsible for the hyper-virulence of the virus during the Hong Kong 1997 outbreak (Claas et al., 1998). MERS-CoV also uses furin to enter the cells (Millet and Whittaker, 2014).

In the case of the influenza viruses, the highly pathogenic forms contain a furin-like cleavage site that can be cleaved by different host proteases allowing the virus to infect a broad range of cells (Kido et al., 2012). The low-pathogenicity forms are only cleaved by trypsin-like proteases and so virus infectivity is limited to the respiratory and/or intestinal organs due to tissue distribution of the activating protease(s) (Sun et al., 2010; Coutard et al., 2020).

Interestingly, a furin-like cleavage site has been identified in the SARS-CoV-2 S protein that is absent in the other SARS-like CoVs and may be responsible for its high pathogenicity (Coutard et al., 2020).

Sequence analysis of the S protein in SARS-CoV-2 reveals the presence of a four amino acid residue insertion; “RRAR”; producing a furin-sensitive motif at the boundary between the S1 and S2 subunits (**Figure 1**). Interestingly this furin cleavage site is conserved among all the isolated and sequenced SARS-CoV-2 virions (Shang et al., 2020a) and its abrogation reduced the efficiency of viral entry (Shang et al., 2020b; Letko et al., 2020).

Processing of the S1/S2 site occurs during viral packaging in infected cells, presumably by furin in the Golgi compartment (Shang et al., 2020b; Letko et al., 2020).

The S protein is further cleaved by host proteases at the S2′ site located immediately upstream of the fusion peptide. This cleavage is proposed to activate the protein for membrane fusion *via* extensive irreversible conformational changes (Letko et al., 2020).

Transmembrane serine protease2 (TMPRSS2) and lysosomal cathepsins both have cumulative effects with furin on activating SARS-CoV-2 entry. However, the furin pre-activation in producer cells allows SARS-CoV-2 to be less dependent on target cell proteases and increases its ability to enter cells with relatively low expressions of TMPRSS2 or lysosomal cathepsins (Shang et al., 2020b; Hoffmann et al., 2020). On the other hand, prior cleavage at the S1/S2 site increases cleavage at the S2′ site (Kuba et al., 2010).

TMPRSS2 activity is important for SARS-CoV-2 cell entry (Braun and Sauter, 2019). The expression of TMPRSS2 and TMPRSS4 in ACE2+ mature enterocytes in the human small intestine facilitates virus entry into these cells. This property makes the intestine a potential site for SARS-CoV-2 infection, which may progress to a systemic disease (Zang et al., 2020).

**Figure 2:**
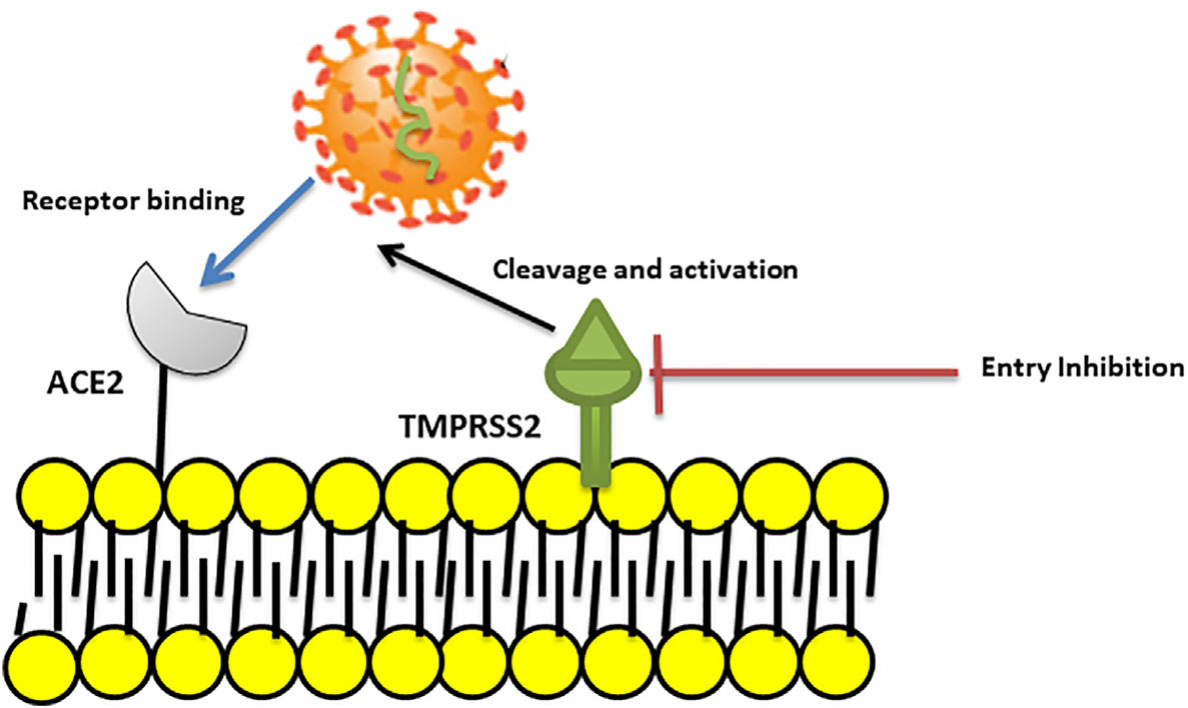
Blocking SARS coronavirus2 (SARS–CoV2) cell entry. SARS-CoV-2 binds to ACE2 and then uses the transmembrane Serine Protease 2 (TMPRSS2) for S protein priming and entry to cell. A TMPRSS2 inhibitor can blocked viral entry by binding to and inhibition of enzyme activity.

It is suggested that “camostat mesylate”, a TMPRSS2 inhibitor approved for clinical use, may block viral entry and could be considered as a potential treatment option (Hoffmann et al., 2020) (**Figure 2**). However, in the case of SARS-CoV, the virus can use endosomal cysteine proteases including cathepsins B and L for S protein priming in TMPRSS2 negative cells (Simmons et al., 2005).

## Mechanism of Viral Cell Entry

A key to preventing the spread of SARS CoV-2 and the resultant pandemic is to gain a clear understanding of its mechanism of cell entry. The virus surface S protein mediates this process by binding to ACE2. Cleavage of the S glycoprotein between the S1 and S2 domains starts during viral packaging. This process is completed by the type II transmembrane serine protease TMPRSS2 (Kreye et al., 2020) which results in activation of the S2 subdomain.

**Figure 3:**
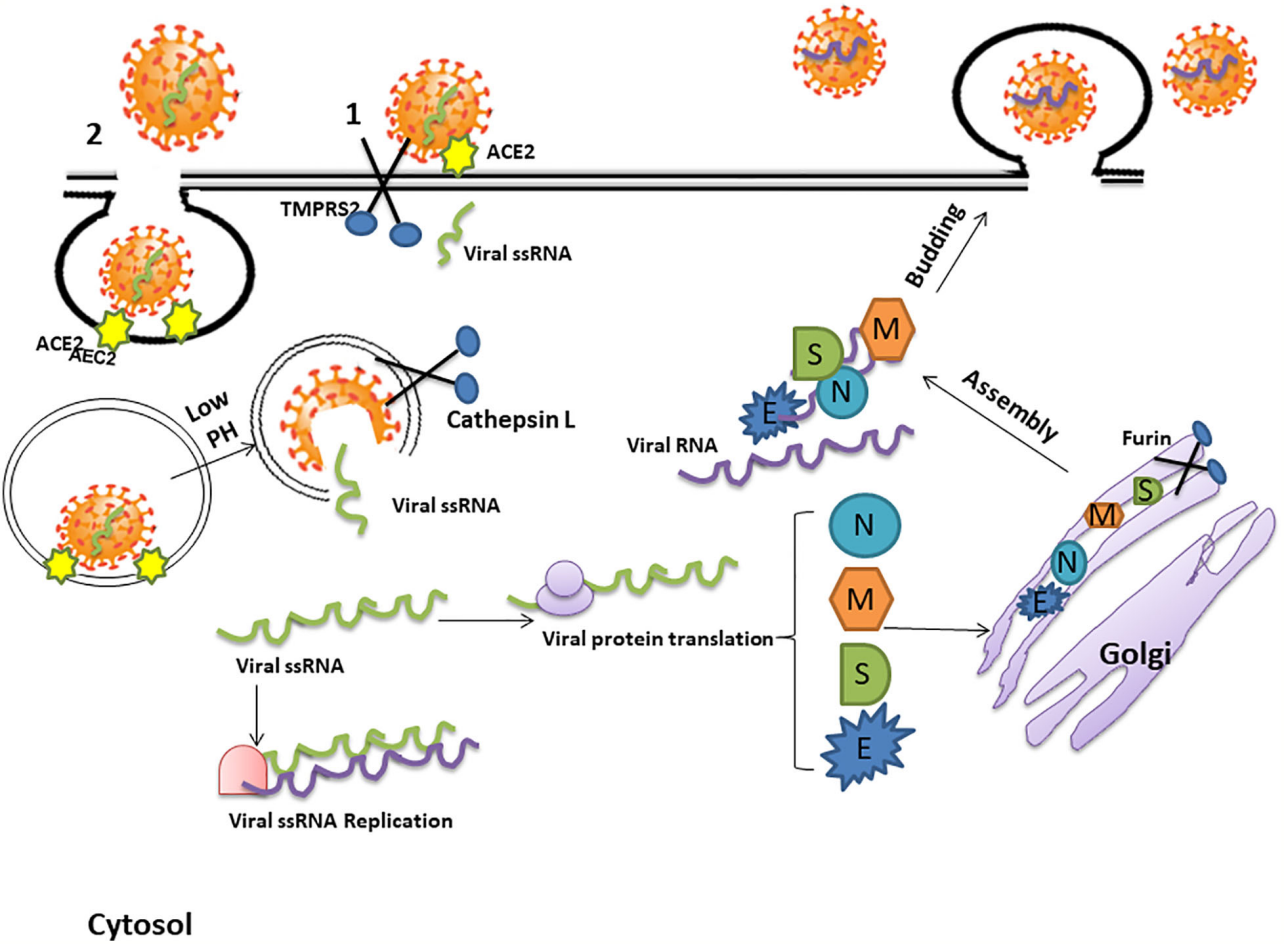
The mechanism of SARS-CoV-2 cell entry and replication. The coronavirus spike (S) protein binds to angiotensin converting enzyme 2 (ACE2) receptors. Cleavage of the S glycoprotein between the S1 and S2 domains, which begins during viral packaging by furin in the Golgi compartments, is completed by the protease trans-membrane serine Protease 2 (TMPRSS2) and lysosomal Cathepsin which enables cell membrane-viral fusion and viral RNA release. This process may either create a pore allowing the viral RNA and RNA-associated proteins to gain access to the cytoplasm (1) or, alternatively, the ACE2/SARS-CoV-2 complex may be internalized by endocytosis and be uncoated in the acidic lysosomal environment to enable release of the single stranded viral RNA (ssRNA) into the cytosol (2). The viral genome is then replicated and translated into viral proteins by the host cell machinery. The newly formed envelope glycoproteins are processed within the Golgi. Further assembling of the genomic RNA and nucleocapsid protein results in the formation of viral particles which are released *via* vesicular exocytosis.

The S2 subdomain then mediates the fusion of the viral genome with the host cell membrane to create a pore allowing the viral RNA and RNA-associated proteins to gain access to the cytoplasm. Another possibility is that ACE2/SARS-CoV-2 complex undergoes endocytosis. The rapid endocytosis of SARS-CoV-2 occurs through clathrin-mediated endocytosis (Millet and Whittaker, 2014). However, it is not completely clear how the viral genome of SARS-CoV-2 gains access to the cytoplasm (**Figure 3**).

However; after entering the cell, the viral RNA is released into the cytoplasm and translates viral proteins followed by viral genome replication (Kuba et al., 2010). The newly formed envelope glycoproteins are inserted into the membrane of the endoplasmic reticulum or Golgi, forming nucleocapsids by assembling the genomic RNA and nucleocapsid protein. Viral particles then incorporate into the endoplasmic reticulum-Golgi intermediate compartment (ERGIC) and the vesicles containing the virus particles fuse with the plasma membrane to release the virus (Chockalingam et al., 2016) (**Figure 3**).

## Immunological Outcome and Pathological Effect of COVID-19

During viral infections, the innate immune system acts as a first line defense to prevent viral invasion or replication. This innate immune response utilizes pattern recognition receptors (PRRs) to detect specific viral components such as viral RNA. Following viral entry into the cell, single stranded RNA viruses are recognized by PRRs such as Toll-like receptor TLR7 and TLR8, RIG-I (retinoic acid-inducible gene I)-like receptors (RLRs), and the NOD-like receptor, CARD-containing-2 (NLRC2), that are expressed by airway epithelial cells and innate immune cells including alveolar and tissue macrophages. RLRs are also able to detect double-stranded (ds) RNA structures (Streicher and Jouvenet, 2019). Sensing of ssRNA by PRRs results in the production of the Type-I and -III antiviral interferons (IFNs) and chemokines. The activation of this IFN-mediated antiviral response is the first major defense mechanism against viral infections (Streicher and Jouvenet, 2019).

Although a rapid and well-coordinated immune response is necessary for a potent defense against viral infection, an excessive inflammatory response may lead to tissue damage at the systemic level. The massive production of cytokines and chemokines detected during COVID-19 infection, the so-called “cytokine storm”, is mainly responsible for the broad and uncontrolled tissue damage observed. The cytokine storm resembles the cytokine release syndrome (CRS) and results in plasma leakage, vascular permeability and disseminated vascular coagulation. These excessive proinflammatory host responses are major factors in the pathological outcomes such as acute lung injury (ALI) and acute respiratory distress syndrome (ARDS) seen in severe SARS-CoV-2 infected patients (Bhaskar et al., 2020).

In addition to the dominant manifestation of respiratory symptoms, some patients have severe cardiovascular damage and individuals with underlying cardiovascular disease (CVD) have an increased risk of death (Zheng Y.Y. et al., 2020). The mechanism underling the acute myocardial injury might be related to the high expression of ACE2 in the CVS (de Abajo et al., 2020).

On the other hand, the combination of cytokine storm together with respiratory dysfunction and hypoxaemia may be a mechanism by which COVID-19 results in damage to myocardial cells (Zheng Y.Y. et al., 2020).

Patients also show lymphopenia and pneumonia with characteristic pulmonary ground-glass opacity changes on chest CT (Wu et al., 2020a; Zhang et al., 2020). Other forms of severity including myocarditis, arrhythmia, cardiogenic shock as well as acute kidney injury have been reported in 10%–30% of ICU patients with confirmed COVID-19 (Murthy et al., 2020). In particular, neurological manifestations (Mao et al., 2020a) and pneumonia were reported in most hospitalized COVID-19 patients (Huang et al., 2020).

### The Pathogenicity of COVID-19 Induced Pneumonia

Among the critically ill patients admitted to ICU, severe disease with respiratory failure due to mucus plugs; severe pneumonia and ARDS is reported in 60%–70% of patients whilst sepsis and septic shock is seen in 30% of patients (Halacli et al., 2020).

SARS-CoV-2 viruses preferentially infect type II epithelial cells within the lungs. In addition, some *in vitro* studies, *ex-vivo*, and in silico studies suggest that mucociliary and goblet cells are also primary target cells for infection (Hui et al., 2020; Sungnak et al., 2020; Mason, 2020). Upon infection, type II epithelial cells undergo programmed cell death as a part of the virus replicative cycle (Anderson et al., 2010). Since these cells are the main player of surfactant production, the reduced surfactant in the alveoli leads the alveoli to collapse which subsequently facilitate pneumonia and ARDS in the severe patients (Ayala-Nunez et al., 2019). In addition, viral infection of the airway epithelial cells can cause high levels of virus-induced pyroptosis with associated vascular leakage. Pyroptosis is a form of programmed cell death which can be triggered by cytopathic viruses (Chen et al., 2019).

In SARS patients, despite a decrease in nasopharyngeal viral titers 10–15 days after the onset of symptoms, the pathological outcomes and alveolar damage continue to worsen (Peiris et al., 2003). This suggests that much of the pathological damage seen is due to the host immune response to infection. It is likely that similar events are occurring with SARS-CoV-2. However, the viral nasopharyngeal load may be very different from that in the lungs as autopsy samples showed a high concentration of virus in different organs including the lung and intestine (Gu et al., 2005; Farcas et al., 2005).

It was also demonstrated that a cytokine storm and its subsequently inflammatory events trigger endothelial activation and induce endotheliitis as well as progressive microvascular pulmonary thrombosis in lung which lead to the disseminated intravascular coagulation (DIC) and impaired lung microcirculation (Luks and Swenson, 2020).

Overall, the main histological findings in the lungs represents patchy necrosis; hyaline membrane formation and hyperplasia of type II pneumocystis that represent diffuse alveolar damage and injury to the gas-exchange surfaces (Anderson et al., 2010). The pathological lung damages in the novel viral disease may be due to either directly viral destruction of alveolar and bronchial epithelial cells or a cytokine storm (Xu Z. et al., 2020). However, respiratory distress in COVID-19 patients may be due to the viral access to CNS and induced damage in the respiratory centers of the brain, making it more complicated to manage these patients (Baig et al., 2020).

### The Neurological Pathogenicity of SARS-CoV-2

36.4% of patients with COVID-19 develop neurological symptoms (Mao et al., 2020a; Mao et al., 2020b) and the neuro-invasive properties of SARS CoV-2 is now accepted.

The retrospective studies as well as case reports from different region in the world; indicate that Covid-19 affects CNS in several ways and leads to a broad spectrum of neurological symptoms from a simple headache to more serious encephalitis (Sheraton et al., 2020).

Autopsy reports have revealed edema and partial neuronal degeneration in brain tissue in severe patients (Channappanavar and Perlman, 2017) and the presence of SARS-CoV-2 RNA in the cerebrospinal fluid (CSF) was confirmed by genome sequencing (Babaha and Rezaei, 2020). However, the pathophysiological characteristics of SARS-CoV-2-associated encephalitis are not fully understood.

The new virus may induces nerve damage through the several mechanisms including direct infection or immune injury (Wu et al., 2020b) which may result in edema and alterations in consciousness (Ye et al., 2020).

Coronaviruses are able to reach to the CNS *via* the synapse connected routes and retrograde transport (Perlman et al., 1990; Ye et al., 2020). In the case of murine models of SARS or MERS-CoVs infection, intranasal infection enables the virus to access the brain *via* the olfactory nerves and rapidly infect specific brain areas such as the brain stem and thalamus (Netland et al., 2008) and cause to critical neuronal histopathological changes in these areas. Since SARS-CoV-2 is mainly spread through the respiratory system, retrograde transport through the olfactory nerve may be the main route for transfer of virus to the central nervous system (CNS) (Mori, 2015; Desforges et al., 2019). The occurrence of loss of smell or hyposmia during the early phase of COVID-19 infections should be taken into consideration for the involvement of the CNS (Baig et al., 2020).

Another pathway proposed for the entry of SARS-CoV-2 into the nervous system is *via* the blood circulation and disruption of the blood-brain barrier (BBB). Noroviruses use different mechanisms for disruption of BBB including direct infection of BBB endothelial cells or enhancing BBB permeability by alteration the expression of matrix metalloproteinases (MMPs) and tight junction proteins (Al-Obaidi et al., 2018). Interestingly, the integrity of the epithelial–endothelial barrier is critically compromised in critical COVID-19 cases (Li H. et al., 2020).

A Trojan horse mechanism has also been proposed to be used by SARS-CoV-2 to reach the CNS (Park, 2020). Here, ACE2-expressing CD68+CD169+ macrophages act as the carrier cell and may contribute to viral spread in COVID-19 patients and induce an enhanced inflammatory response during SARS-CoV-2 infection (Park, 2020).

The highly specialized functions and limited regenerative capacity of neurons means that chronic and latent CNS SARS-CoV-2 infection may have long-term detrimental consequences (Andries and Pensaert, 1980). SARS-CoV-2 could trigger CNS degeneration and may be a potential trigger of future neurodegenerative diseases such as Parkinson’s disease or multiple sclerosis (Serrano-Castro et al., 2020). Considering the large number of patients involved globally, the risk of future neurological disorders is worrying and the clinicians should pay more attention to the neurologic symptoms in COVID-19 patients especially in the early phase of infection.

## Cellular and Molecular Mechanisms of Disease

Based on data from the hospitalized patients, the majority of COVID 19 cases (about 80%) are asymptomatic or show mild symptoms while the rest experience severe respiratory failure (Surveillances, 2020). This may be explained by the fact that the onset and development of COVID-19 depends upon virus infectivity and the strength of the individual’s immune response. The viral factors include virus type, titer, viability and mutations, whilst the host immune factors including age, gender and genetics (such as HLA genes) which together determine the duration and severity of the disease (Rithanya and Brundha, 2020).

SARS-CoV-2 tends to have a long incubation period: 5–15 days on average. This long incubation period is due to its ability to escape from host immune detection at the early stages of infection (Prompetchara et al., 2020). At the initiation of infection, inhaled SARS-CoV-2 binds to ACE2 on nasal epithelial cells and starts replicating. Despite the low viral burden and local propagation of virus, the virus can be detected by nasal swabs by RT-PCR (Mason, 2020). There is a low immune response at this step. The virus then replicates and migrates through the conducting airways and a more robust innate immune response is triggered. At this time, the disease COVID-19 clinically manifests itself and early markers of the innate immune response such as CXCL10 are detectable (Mason, 2020).

CXCL10 is known as a disease marker in SARS (Xu Z. et al., 2020) and its expression is significantly increased in alveolar type II cells in response to both SARS-CoV (Qian et al., 2013) and to influenza (Wang J. et al., 2011). Higher levels of interleukin IL-6 and IL-10, and lower levels of CD4+T and CD8+T are also observed in patients with COVID-19 and these correlate with the severity of disease (Wan S. et al., 2020).

In the remainder of the review, we discuss the potential mechanisms by which SARS-CoV-2 modulates the host immune system predominantly based on the strategies used by SARS-CoV and MERS.

### Innate Immune Responses

The innate immune response is the first line of defense against viral infection and has a determinant role in protective or destructive responses upon infection (Kindler et al., 2016). Upon viral infection, type I interferon (IFN) responses and its downstream cascade are initiated in order to control viral replication and induce an effective adaptive immune response (Prompetchara et al., 2020).

**Figure 4:**
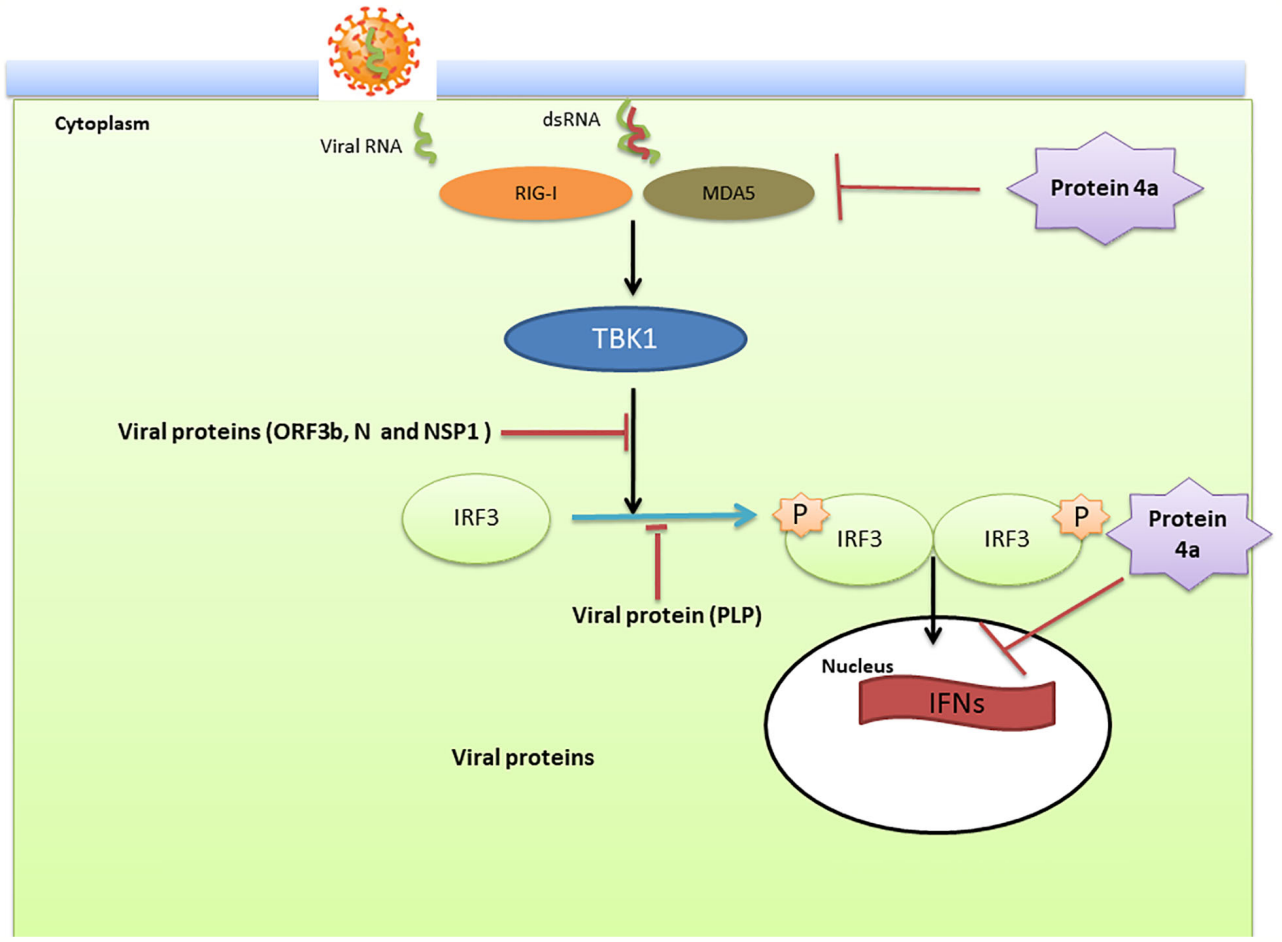
The innate immune modulation mechanisms by SARS-CoV and Middle East respiratory syndrome-related coronavirus (MERS-CoV). Double stranded (ds)RNA, a by-product of RNA virus replication in the cytoplasm, is sensed by the pattern recognition receptors such as retinoic-acid inducible gene I (RIG-I) and melanoma differentiation-associated protein 5 (MDA5) which subsequently leads to activation of the kinase TANK Binding Kinase 1 (TBK1). These kinases then phosphorylate interferon (IFN) regulatory factor 3 (IRF3) and traffic to the nucleus to active the transcription of IFNs. Viral proteins actively modulate this pathway. Open reading frame (ORF)3b, nucleocapsid (N), and non-structural protein (NSP)1 affect the signal transduction pathway that activates IRF3. In addition, the papain-like protease (PLP) blocks the phosphorylation of IRF3 and its activation. Viral protein 4a suppress the activation of RIG-I/MDA5 signaling and blocks the induction of IFNs through interaction with dsRNA.

In the case of SARS-COV and MERS; the virus modulate anti-viral IFN responses by using different strategies that interfere with IFN production and its downstream signaling pathways (Kindler et al., 2016). This dampening procedure is closely associated with disease severity. In two MERS-CoV patients with different severities, the type I IFN response was significantly lower in the patient with a poor outcome (death) than in the patient who recovered (Shokri et al., 2019). IFN production requires the phosphorylation and activation of IFN-regulatory factor3 (IRF3) which is inhibited following infection with SARS-CoV (Kindler et al., 2016) (**Figure 4**).

Nuclear translocation of IRF3 and its subsequent stimulation of IFN β expression is inhibited by viral ORF4a, ORF4b, and ORF5 proteins (Yang et al., 2013; Yang Y. et al., 2015). In addition, the MERS-CoV accessory protein 4a blocks IFN induction through direct interaction with double-stranded RNA (Niemeyer et al., 2013). Based on the genomic similarity between SARS-CoV-2, SARS-CoV and MERS-CoV it is likely that SARS-CoV-2 utilizes similar strategies to modulate the type I IFN response (Dandekar and Perlman, 2005).

However, sequence analysis showed several changes to SARS-CoV-2 that indicate that SARS-CoV-2 is more sensitive to type I IFN (Lokugamage et al., 2020). ORF3b from SARS-CoV encodes a 154 amino acid (aa) protein that suppress the type I IFN responses by inhibition of IRF3 phosphorylation. In SARS-CoV-2 a premature stop codon in ORF3b results in a truncated 20aa protein which lacks an equivalent function to that seen with SARS-CoV (Lokugamage et al., 2020). SARS-CoV-2 has similar viral replication kinetics to SARS-CoV in Vero cells (Lokugamage et al., 2020) but is much more sensitive to type I IFN pre-treatment and a significant reduction in viral replication is seen following type I IFN treatment (Lokugamage et al., 2020b). Together these observations suggest that type I IFN may be a potential treatment for COVID-19 as seen in animal models of SARS-CoV and MERS-CoV infection (Channappanavar et al., 2019). In contrast, a delayed induction of type I IFN compromises the early viral control and drives the influx of hyper-inflammatory neutrophils and monocytes-macrophages which lead to a mass production of pro-inflammatory cytokines and may evoke a cytokine storm (Channappanavar and Perlman, 2017).

A cytokine storm leads to an uncontrolled systemic inflammatory response that may trigger a severe attack on the body by its own immune system. As with SARS-CoV and MERS-CoV infection, this may lead to ARDS, multiple organ failure and death in severe cases of SARS-CoV-2 infection (Channappanavar and Perlman, 2017; Tabarsi, 2020). In severe COVID-19 patients, serum levels of proinflammatory cytokines including IL-2, IL6, IL-7, IL-10, G-CSF, IP-10, MCP-1, MIP-1α and TNFα are highly elevated (Xu Z. et al., 2020). Furthermore, increased total blood neutrophils and decreased total blood lymphocytes in patients within ICU compared with patients not in ICU care correlated with disease severity and death (Prompetchara et al., 2020).

The previous studies and clinical evidence also strongly indicate the importance of the NLR family pyrin domain containing 3 (NLPR3) inflammasome in the pathologic effects of severe COVID-19 (Shah, 2020). In patients with reduced immune fitness whose innate immune system is unable to clear the virus, NLRP3 activation may occur (Freeman and Swartz, 2020). A sustained and unregulated NLRP3-dependent inflammatory response leads to the severe clinical symptoms including necrosis, fever, release of damage-associated molecular patterns (DAMP) and severe inflammation (van den Berg and Te Velde, 2020). Direct activation of NLRP3 induces pyroptosis and cell death in human cells. Additionally, hyper-activation of NLRP3 may result in coagulopathy, neutrophil infiltration, Th17 and macrophage activation and a cytokine storm in severe COVID-19 patients (Merad and Martin, 2020). Genetic variations in host inflammasome pathways may also influence disease outcome and may be responsible for the heterogeneous response of patients with COVID-19 and the array of clinical severity (Freeman and Swartz, 2020).

### Adaptive Immune Responses

Corona viruses including SARS and MERS have ability to infect immune cells which plays a key role in the disease pathogenesis (Dandekar and Perlman, 2005). In addition, MERS-CoV induces rapid apoptosis of macrophages by the limiting of the early induction of IFN (Shokri et al., 2019). In the case of SARS–CoV, infection of lymphocytes has been proposed to play the major role in viral-induced pathogenicity. SARS-CoV-infected lymphocytes, similar to feline infectious peritonitis virus (FIPV)-infected macrophages in domestic cats, might transport the virus to distant organs resulting in systemic infection (de Groot-Mijnes et al., 2005).

Upon virus entry, immunogenic peptides are presented to T cells in association with human leukocyte antigen (HLA) on the surface of antigen presenting cells (APC). Polymorphisms in the HLA system may influence the susceptibility and outcome of SARS-CoV infection (Yuan et al., 2014). Some HLA alleles including HLA-B*4601, HLA-B*0703, HLA-DRB1*1202 HLA-DRB4*01010101, and HLA-Cw*0801 are associated with susceptibility to SARS-CoV infection (Wang S.-F. et al., 2011). Whereas, the HLA-DR0301, HLA-Cw1502, and HLA-A*0201 alleles are protective against severe SARS infection (Hajeer et al., 2016). Furthermore, polymorphisms within the MBL (mannose-binding lectin) gene are also associated with susceptibility to SARS-CoV infection (Tu et al., 2015).

In the case of MERS-CoV infection, HLA-DRB1*11:01 and HLA-DQB1*02:0 alleles increased the risk of infection (Wang S-F. et al., 2011). These alleles should be assessed in COVID-19 patients (Li X. et al., 2020). In addition, antigen presentation *via* the class I and II major histocompatibility complex (MHC) was down-regulated in MERS-CoV infection. This would markedly diminish T cell activation in response to the virus (Shokri et al., 2019). During viral infection, antigen presentation triggers both humeral and cellular immunity mediated by virus-specific B and T cells.

#### B Cell Responses

B cell profiling by RNA-seq showed a specific pattern of new B cell-receptor changes (IGHV3–23 and IGHV3–7) in COVID-19 patients. It is also indicated that the number of naïve B cells is decreased while the number of peripheral blood plasma cells was remarkably increased (Wen et al., 2020).

With SARS infection, the profile of neutralizing antibodies are predominantly IgM and IgG. Specific B and T cells epitopes are commonly mapped against the structural S and N proteins (Berry et al., 2010; Liu et al., 2017). SARS-CoV infection induces seroconversion 4 days after the onset of disease and SARS-specific IgM antibodies are found in most patients up to week 12 post-infection while the IgG antibody can persist for much longer. Indeed, long lasting specific IgG was detected up to 2 years post infection (Li et al., 2003).

In COVID-19, SARS-CoV-2 specific antibodies may be produced to neutralize the virus. In one patient, serology reports showed the peak of specific IgM at day 9 after the onset of the disease and switching to IgG by week 2 (Zhou P. et al., 2020). Interestingly, sera from five patients with confirmed COVID-19 show some cross-reactivity with SARS-CoV. Furthermore, all sera from patients were able to neutralize SARS-CoV-2 in an *in vitro* plaque assay, suggesting a possible successful mounting of the humoral responses (Zhou P et al., 2020).

In a recent study of 285 patients with mild to severe disease, 96.8% of tested patients achieved seroconversion of IgG or IgM within 20 days after the onset of symptoms with the titer plateauing within 6 days after seroconversion. Moreover, 100% of patients had positive virus-specific IgG approximately 17–19 days after symptom onset. In addition, 94.1% patients were positive for virus-specific IgM approximately 20–22 days after symptom onset (Liu et al., 2020; Long et al., 2020). Antibody titers in the severe group were higher than those with milder disease. Interestingly, a number of individuals with negative nucleic acid results and no symptoms had positive IgG and/or IgM tests (Long et al., 2020). This highlights the importance of serological testing as a complement to the RT-PCR test in surveying for asymptomatic patients in close contact with other (Liu et al., 2020; Long et al., 2020).

#### T Cell Responses

During viral infection, T helper (Th) cells play an important role in the adaptive immunity. The cytokine microenvironment generated by antigen presenting cells directs T cell responses. In SARS-CoV infection a strong Th1 cell response and higher levels of neutralizing antibodies were observed in the mild-moderate group, while in the fatal group a higher level of Th2 cytokines (IL-4, IL-5, IL-10) was detected (Li et al., 2008). Current evidence indicates that a Th1 response is crucial for the successful control of SARS-CoV and MERS-CoV and this may also be essential for SARS-CoV-2 (Li et al., 2008).

The balance between naïve and memory T cells is crucial to controlling infection. Naïve T cells are responsible for the defense against new, previously unrecognized infection by the production of cytokines. In contrast, memory T cells promote antigen-specific immune responses. Disruption of the balance in favor of naïve T cells could strongly promote hyper inflammation. On the other hand, the reduction in memory T cells could contribute to COVID-19 relapse, which is reported in a number of recovered cases of COVID-19 (Zhou L. et al., 2020; Yuan et al., 2020; Chen D. et al., 2020).

In severe patients with COVID 19, CD8+ T cell responses were more frequent and robust than CD4+ T cell responses and an early rise in CD8+ T cell numbers was correlated with disease severity (Prompetchara et al., 2020). CD8+ T cells contained a large number of cytotoxic granules which may have induced severe immune injury in the patients (Xu Z. et al., 2020). Additionally, a recent report of a 50 year old male with covid-19 who died showed a significantly reduced number of peripheral blood CD4+ and CD8+ T cells. These cells had a hyper-activated appearance and were double positive for HLA-DR and CD38 (Xu Z. et al., 2020).

COVID-19 also induces T-cell exhaustion (Diao et al., 2020). T cell exhaustion is a state of T cell dysfunction that arises during many chronic infections as well as during persistent viral infections (Wherry, 2011). Peripheral blood T cells from COVID-19 patients expressed high levels of the exhaustion markers PD-1 and Tim-3 (Diao et al., 2020).

Peripheral blood total counts of T cells, CD4+, and CD8+ T cells were significantly lower in ICU patients than non-ICU cases with COVID-19 and counts were negatively correlated with patient survival (Diao et al., 2020). It seems that the cytokine storm may promote apoptosis or necrosis of T cells and thereby reduce their numbers (Xi-zhi and Thomas, 2017). Furthermore, the levels of TNF-α, IL-6, and IL-10 were significantly increased in infected patients and raised even more in patients who required ICU treatment. Interestingly, the concentrations of these cytokines were negatively correlated with total T cell counts (Diao et al., 2020).

Besides suppression of T cell proliferation, IL-10 can induce T cell exhaustion by increased levels of PD-1 and Tim-3 (Brooks et al., 2006). Inhibition of IL-10 also reduced the degree of T cell exhaustion in animal models of chronic infection (Ejrnaes et al., 2006). TNF-α is a pro-inflammatory cytokine that promotes T cell apoptosis *via* the TNF receptor TNFR-1 signaling pathway. The expression of TNFR-1 is increased in aged T cells (Aggarwal et al., 1999). IL-6 contributes to host defense by stimulating acute phase responses or immune reactions in response to infections and tissue injury. Deregulated and continual synthesis of IL-6 plays a pathological role in chronic inflammation and infection (Jones and Jenkins, 2018). The level of IL-6 in severe patients continue to increase over time and is higher in non-survivors (Zhou F et al., 2020).

It is suggested that the different mortality rates in COVID-19 patients is due to the difference in the response to infection (Bienvenu et al., 2020). Analysis of viral RNA in COVID-19 patients indicated that the males show delayed viral clearance (Xu K. et al., 2020; Zheng S. et al., 2020). Furthermore, it is reported that male patients had higher plasma levels of innate immune cytokines including IL-8 and IL-18 along with activated non-classical monocytes. In contrast, female patients had a more robust T cell activation during SARS-CoV-2 infection. A poor T cell response may be responsible for the worse outcome in male patients, while in female patients, higher levels of innate immune cytokines were associated with worse disease (Takahashi et al., 2020).

## Covid-19 and Post Infection Immunity

Another question regarding this new disease is that whether the infection induces persistent immune memory that could protect the recovered individual against reinfection. The durability of protective antibodies induced by SARS-CoV-2 or the antibody titers that will protect against reinfection is still unclear. It is reported that antibodies disappear rapidly after recovery particularly in patients with mild disease. For example, IgG has a half-life of approximately 21 days in SARS-CoV-2 patients. However; even in the absence of specific serum antibodies, the presence of memory B and T cells may be maintained (Cox and Brokstad, 2020).

In a recent study, COVID-19 induced memory lymphocytes with an antiviral protective immune function. This longitudinal study assessed the immune response in patients who recovered from mild symptomatic COVID-19. These recovered individuals developed SARS-CoV-2-specific IgG antibody and neutralizing plasma in addition to virus-specific memory B and T cells that had ability to expand over three months following the onset of symptoms (Rodda et al., 2020). Furthermore, following antigen re-exposure the memory T cells secreted IFN-γ and were able to clonally expand whilst memory B cells expressed antibodies capable of neutralizing the virus (Rodda et al., 2020).

Studies of patients infected with SARS-CoV in 2003 suggested that the infection induced durable T cell responses lasting for up to 6 years but no prolonged memory B cells. Importantly, these T cells could cross-react with the SARS-CoV-2 virus after 17 years, but it is not clear that whether they can provide protection against COVID-19 (Le Bert et al., 2020). On the other hand, recent reports demonstrated that T cell reactivity against SARS-CoV-2 exists in many unexposed people. It is hypothesized that this might be due to immunity to common cold coronaviruses that could influence COVID-19 disease severity (Sette and Crotty, 2020).

## Potential Pharmacologic Strategies in the Treatment and Protection

At present, there are no specific antiviral drugs or a vaccine available to treat COVID-19. Currently, broad-spectrum antiviral drugs like nucleoside analogues and HIV-protease inhibitors are being used to attenuate viral infection (Lu, 2020). Other treatment regimens include a combination of oral oseltamivir, lopinavir and ritonavir and intravenous administration of ganciclovir for 3–14 days (Chen N. et al., 2020). There is an ongoing clinical trial for examination of the safety and efficacy of lopinavir-ritonavir and IFN-2b in patients with COVID-19 (Lu, 2020; Habibzadeh and Stoneman, 2020). Although the antivirals remdesivir and chloroquine controlled SARS-CoV-2 infection *in vitro*, these have shown variable results in the clinic (Wang et al., 2020). EIDD-2801 is a new drug used to control and treat seasonal and pandemic influenza virus infections. It has high therapeutic efficacy in humans and may be considered as a potential drug for the treatment of COVID-19 infection (Toots et al., 2019).

Therapies based on the ACE2 receptor have raised concerns about the use of Renin-Angiotensin-Aldosterone System (RAAS) inhibitors that may alter ACE2 function and expression. Angiotensin II receptor blockers (ARBs) and other RAAS inhibitors increased ACE2 expression in the lung in animal models (Gurwitz, 2020). Data from cohort and cross sectional studies in patients with heart failure or cardiovascular disease showed that the effect of these inhibitors on ACE2 is not uniform even in response to a given drug class (Shearer et al., 2013). In addition, recent evidence suggests that ACE inhibitors do not affect COVID-19 infection or severity (Bryan Williams, 2020). Angiotensin receptor 1 (AT1R) blockers, such as losartan, have also been suggested as a therapeutic approach for reducing the severity and mortality of SARS-CoV-2 infections (Gurwitz, 2020).

Interestingly, human recombinant soluble ACE2 (hrsACE2) blocked SARS-CoV-2 infections in engineered human tissues, which suggests promise as a treatment capable of stopping early infection of the novel coronavirus. In this regard, APN01 developed by APEIRON is currently in clinical trials (Zoufaly et al., 2020). SARS-CoV-2 uses the serine protease TMPRSS2 for S protein priming. It is suggested that previously approved TMPRSS2 inhibitors such as nafamostat mesylate and camostat mesylate (Hoffmann et al., 2020) will block viral entry and may be a possible treatment option (Tay et al., 2020).

The central role of cytokine dysregulation in the pathogenicity of seriously ill COVID-19 patients has indicated the potential of cytokine-targeted therapy in managing disease progression (Rahmati M. 2020). Drugs such as IL-6 inhibitors (tocilizumab, sarilumab, and siltuximab) or IL1-β inhibitors are possible interventions in this area.

Tocilizumab (Actemra) is a humanized anti-IL-6 receptor antibody that was successful in the treatment of rheumatoid arthritis (RA) and juvenile idiopathic arthritis (Burmester et al., 2016; Yokota et al., 2016). Whether tocilizumab restores T cell counts in COVID-19 patients by suppressing IL-6 signaling needs to be investigated (Diao et al., 2020; Atal and Fatima, 2020). Interleukin-1 targeting with a high-dose of anakinra in patients with COVID-19 was safe and showed clinical improvement in 72% of patients in a retrospective cohort study (Cavalli et al., 2020). Anakinra is a recombinant interleukin-1 (IL-1) receptor antagonist that has anti-inflammatory and immunomodulatory effect used to treat of inflammatory arthritides (Furst, 2004).

The NLRP3 inflammasome with its downstream pathways is also an attractive target for therapy of COVID-19 (Pontali et al., 2020; Jamilloux et al., 2020; Soy et al., 2020). The first clinical trial study of using tranilast (an NLRP inflammasome inhibitor) to treat COVID-19 is ongoing and registered in the Chinese clinical trial registry (http://www.chictr.org.cn/showprojen.aspx?proj=49738).

SARS-CoV-2 is more sensitive to type I IFN *in vitro* and *in vivo* than SARS suggesting that type I IFNs may be a potential treatment for protection against COVID19. Interestingly, Bacillus Calmette–Guérin (BCG) vaccination may reduce the mortality associated with COVID-19. BCG vaccine stimulates the production of IFNs by the innate immune system in a TLR2-dependent manner (Ades, 2014; Kativhu and Libraty, 2016) and may provide protection against COVID19. IFN-β may prove efficacious as a vaccine adjuvant to boost immune cell function and INF production (Ades, 2014).

Remdesivir an antiviral compound that was firstly introduced for Ebola virus (Wang et al., 2020) and was successful in shortening the time to recovery in adults hospitalized with COVID-19 (Lu, 2020; Beigel et al., 2020; Harapan et al., 2020). There are ongoing clinical trials in a number of countries for remdesivir as a potential treatment for COVID-19. Remdesivir, made by Gilead, is currently going through the FDA approval process and is authorized in the United States for use under an Emergency Use Authorization (EUA). Currently, the drug is given intravenously through daily infusions in the hospital. However, an inhaled formulation given through a nebulizer, may potentially allow for easier administration outside the hospital at earlier stages of the disease.

These approaches would be valuable to investigate for COVID-19 and open a window for finding new ways to protect against and treat this deadly epidemic. There are currently 109 ongoing clinical trials for COVID-19 registered on clinicaltrials.gov. These include numerous pharmacological and biological approaches and the use of natural products.

## Conclusion

The COVID-19 pandemic is rapidly spreading across the world and has caused more deaths compared with SARS or MERS. Despite the high rate of mortality and morbidity, no medication or vaccine has been consistently shown to be effective. SARS-CoV-2 belongs to the Coronaviridae family which was also responsible for previous widespread outbreaks of SARS‐CoV in 2002 and MERS‐CoV in 2008. The rapid spread, induction of severe infection, cross-species transmission and unpredicted behavior of coronaviruses result in them being a continuous threat to human health. This is important due to the existence of many animal reservoirs for CoVs and the lack of an approved treatment.

There is an imperative need to design and develop effective therapeutic and preventive strategies. This will require using the accumulated knowledge of how the host immune response system responds to SARS and MERS but importantly will require analysis of immune responses within the lungs of COVID-19 patients. Techniques such as single cell sequencing and cellular indexing of transcriptomes and epitopes by sequencing (CITE-seq) will tremendously increase our understanding of disease pathology and enable knowledge-based decisions for the adoption of new therapeutic immune modalities.

## Author Contributions

SA wrote first draft. EM, HJ, PT, IMA, MV, and HB have revised equally the first draft. All authors contributed to the article and approved the submitted version.

## Funding

IMA is financially supported by the British Heart Foundation (PG/14/27/30679), the Dunhill Medical Trust (R368/0714), the Welcome Trust (093080/Z/10/Z), the EPSRC (EP/T003189/1), the Community Jameel Imperial College COVID-19 Excellence Fund (G26290) and by the UK MRC (MR/T010371/1).

## Conflict of Interest

The authors declare that the research was conducted in the absence of any commercial or financial relationships that could be construed as a potential conflict of interest.
